# Andrographolide attenuates epithelial‐mesenchymal transition induced by TGF‐β1 in alveolar epithelial cells

**DOI:** 10.1111/jcmm.15665

**Published:** 2020-07-24

**Authors:** Jingpei Li, Jun Liu, Weifeng Yue, Ke Xu, Weipeng Cai, Fei Cui, Zhuoyi Li, Wei Wang, Jianxing He

**Affiliations:** ^1^ Department of Thoracic Surgery The First Affiliated Hospital of Guangzhou Medical University Guangzhou China; ^2^ State Key Lab of Respiratory Diseases Guangzhou Institute of Respiratory Health The First Affiliated Hospital of Guangzhou Medical University Guangzhou China

**Keywords:** alveolar epithelial cells, andrographolide, EMT, oxidative stress, Sirt1/FOXO3 signalling, TGF‐β1

## Abstract

Andrographolide (Andro), a component from Chinese medicinal herb *Andrographis paniculata*, could alleviate pulmonary fibrosis in rodents. Yet, whether and how Andro mitigates epithelial‐mesenchymal transition (EMT) induced by TGF‐β1 remain unknown. This study aimed to explore the effect of Andro on TGF‐β1‐induced EMT in human alveolar epithelial cells (AECs) and the mechanisms involved. We illustrated that Andro inhibited TGF‐β1‐induced EMT and EMT‐related transcription factors in alveolar epithelial A549 cells. Andro also reduced TGF‐β1‐induced cell migration and synthesis of pro‐fibrotic factors (ie CCN‐2, TGF‐β1), matrix metalloproteinases (ie MMP‐2, MMP‐9) and extracellular matrix (ECM) components (ie collagen 1), implying the inhibiting effect of Andro on TGF‐β1‐induced EMT‐like cell behaviours. Mechanistically, Andro treatment not only repressed TGF‐β1‐induced Smad2/3 phosphorylation and Smad4 nuclear translocation, but also suppressed TGF‐β1‐induced Erk1/2 phosphorylation and nuclear translocation in A549 cells. And treatment with ALK5 inhibitor (SB431542) or Erk1/2 inhibitors (SCH772984 and PD98059) remarkably reduced EMT evoked by TGF‐β1. In addition, Andro also reduced TGF‐β1‐induced intracellular ROS generation and NOX4 expression, and elevated antioxidant superoxide dismutase 2 (SOD2) expression, demonstrating the inhibiting effect of Andro on TGF‐β1‐induced oxidative stress, which is closely linked to EMT. Furthermore, Andro remarkably attenuated TGF‐β1‐induced down‐regulation of sirtuin1 (Sirt1) and forkhead box O3 (FOXO3), implying that Andro protects AECs from EMT partially by activating Sirt1/FOXO3‐mediated anti‐oxidative stress pathway. In conclusion, Andro represses TGF‐β1‐induced EMT in AECs by suppressing Smad2/3 and Erk1/2 signalling pathways and is also closely linked to the activation of sirt1/FOXO3‐mediated anti‐oxidative stress pathway.

## 1 INTRODUCTION

Pulmonary fibrosis (PF) is one of chronic maladies, which is characterized by an extravagant accumulation of extracellular matrix (ECM), causing scarring and damage of lung architecture, thereby limiting gas exchange.^1^ Eventually, most of the patients with PF die of irreversible deterioration of pulmonary function.^2^ Now, the occurrence of PF around the world is gradually increasing and the median survival is probably 3‐5 years.^3^ Hence, the treatment of PF remains a major issue to be addressed.

Epithelial‐mesenchymal transition (EMT) is a critical pathological process in PF.^4^, ^5^ It is characterized by numerous molecular and pathophysiological alterations influencing cell phenotype and behaviour, during which epithelial cells gradually lose the original characteristics and have mesenchymal properties.^6^ EMT can be induced within alveolar epithelial cells (AECs) by hypoxia, reactive oxygen species (ROS) and transforming growth factor (TGF)‐β1.^7^, ^8^ Among these factors, TGF‐β1, mainly secreted by epithelial cells, fibroblasts and macrophages, is markedly up‐regulated in the lung tissues of PF patients and is the most latent inductor of EMT during PF progression. Both TGF‐β1‐treated primary AECs and alveolar epithelial A549 cells were observed a transition from epithelial cell morphology to a mesenchymal cell phenotype.^5^, ^9^ TGF‐β1‐induced EMT is largely regulated by Smad‐dependent or Smad‐independent pathways.^10^, ^11^ Hence, inhibition of TGF‐β1‐dependent EMT process may be a promising treatment strategy for PF.

**FIGURE 1:**
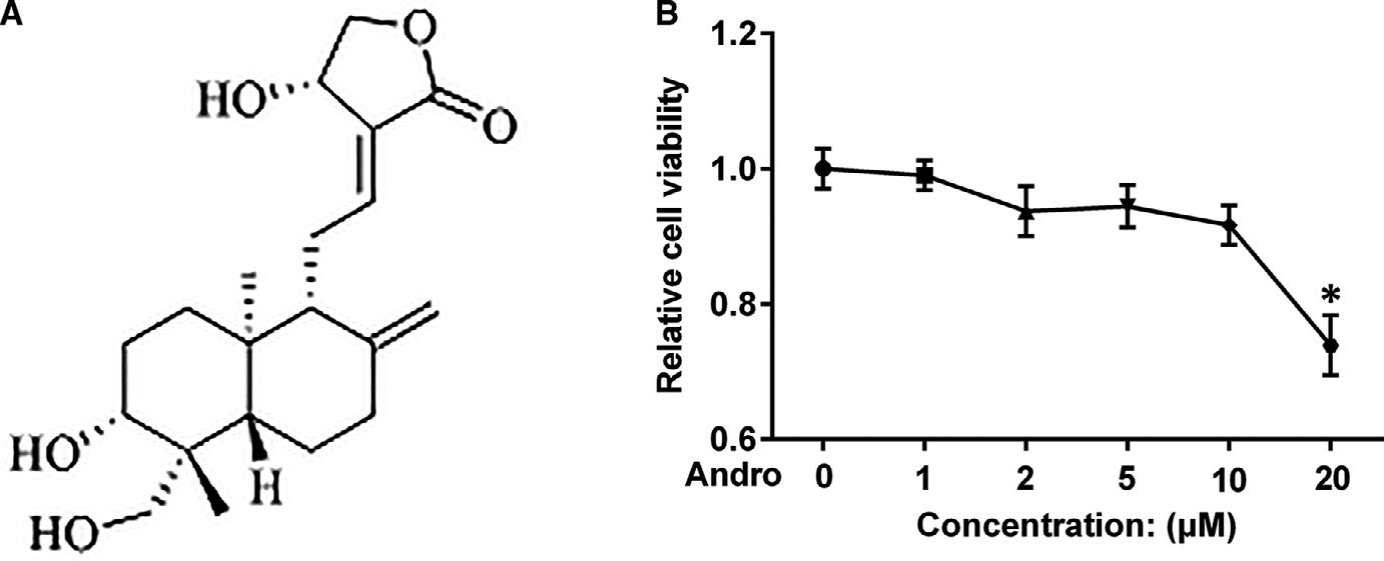
Chemical structure and the effect of Andro on A549 cell viability. (A) Chemical structure of Andro. (B) A549 cells were treated with the indicated concentrations of Andro (0, 1, 2, 5, 10 and 20 μM) for 24 h. The cell viability was measured with CCK‐8 assay. *P < 0.05 vs control [Andro (0)]

Andrographolide (Andro) (Figure 1A) is a diterpenoid derived from *Andrographis paniculata*, which has been shown to have numerous bioactivities such as anti‐inflammatory, antioxidant and anti‐EMT properties.^12^, ^13^, ^14^ Recently, Andro was found to inhibit bleomycin (BLM)‐induced PF in rodents by inhibiting inflammatory response, oxidative stress, fibroblast proliferation and differentiation.^15^, ^16^, ^17^ Andro was also found to mitigate silica‐induced PF by inhibiting inflammation, oxidative stress and EMT phenotype.^18^ Nevertheless, the direct effects of Andro on the TGF‐β1‐induced EMT within AECs remain unknown.

The goal of this study was to explore the direct effects of Andro on the TGF‐β1‐induced EMT in AECs and explore the feasible molecular mechanisms. We illustrated that Andro ameliorated TGF‐β1‐induced EMT in AECs through suppressing Smad2/3 and Erk1/2 signalling pathways. Andro also activated Sirt1/FOXO3‐mediated anti‐oxidative stress pathway to suppress EMT in AECs.

## 2 MATERIALS AND METHODS

### 2.1 Chemicals and reagents

Andro was purchased from Chengdu Herbpurify Co., Ltd (Chengdu, China). Recombinant TGF‐β1 was purchased from Novoprotein (SinoBio, Shanghai, China). N‐Acetyl‐l‐cysteine (NAC) was purchased from Sigma‐Aldrich (St Louis, MO, USA). SCH772984, PD98059 and SB431542 were purchased from Selleck (Houston, TX, USA). The TRIzol reagent was purchased from Invitrogen (Carlsbad, CA, USA). The PrimeScript RT reagent Kit with gDNA Eraser was obtained from Takara Bio Inc (Shiga, Japan), and the SsoFast EvaGreen Supermix was obtained from Bio‐Rad Laboratories Inc. (Hercules, CA, USA). Antibodies to sirtuin1 (Sirt1), E‐cadherin, p‐Smad2, Smad2, p‐Smad3, Smad3, p‐Erk1/2, Erk1/2, N‐cadherin, snail, slug and vimentin were from Cell Signaling Technology (Beverly, MA, USA). Antibodies to fibronectin, matrix metalloproteinase (MMP)‐2, MMP‐9 and TGF‐β1 were from Proteintech (Chicago, IL, USA). Antibodies to α‐SMA were from Sigma‐Aldrich. Antibodies to NADPH oxidase 4 (NOX4), superoxide dismutase (SOD) 2, Forkhead box O3 (FOXO3) and GAPDH were purchased from ABclonal (Wuhan, China); all the secondary antibodies were from Abcam Biotechnology (Cambridge, MA, USA). All other chemicals and reagents used in the experiment were of analytical grade. Other reagents were all from Beyotime Institute of Biotechnology unless otherwise indicated.

### 2.2 Cell culture

Human A549 cells were bought from the Cell Bank of the Chinese Academy of Sciences (Shanghai, China) and grew in DMEM (Gibco, NY, USA) with 10% FBS (Biochrom, Berlin, Germany), 100 KU/L penicillin and 100 mg/L streptomycin at 37°C in 5% CO_2_.

### 2.3 Morphological analysis

A549 cells were seeded into a six‐well plate and incubated with 2, 5 and 10 μM of Andro in the presence and absence of TGF‐β1 (5 ng/mL; Novoprotein) for 24 h. Cell morphology was randomly captured with an inverted microscope (Leica, Wetzlar, Germany).

### 2.4 Cell viability analysis

A549 cells were cultured in a 96‐well plate and incubated with different concentrations of Andro (2, 5, or 10 μM). Cell viability was measured with a CCK‐8 Kit (Dojindo, Kumamoto, Japan).

### 2.5 Immunofluorescence staining

Immunofluorescence staining was performed as described previously.^17^ Briefly, A549 cells were seeded on glass slides in 12‐well plates and incubated as indicated for 24 hours. The slides were washed three times with PBS, fixed in 4% paraformaldehyde for 10 minutes and then permeabilized with 0.1% Triton X‐100 for 20 minutes. Whereafter, the cells were blocked with 3% BSA at room temperature for 1 hour and subsequently incubated with primary antibody (vimentin, 1:100; p‐Erk1/2, 1:100) at 4°C overnight. After incubation, slices were washed three times with PBS and incubated with corresponding secondary antibodies (1:200; Beyotime Institute of Biotechnology, Haimen, China). The nuclei were stained with DAPI (Beyotime Institute of Biotechnology) for 5 minutes. Samples were washed three times with PBS and mounted in antifade mounting medium, and fluorescence was detected using a confocal laser scanning microscope (Nikon D‐Eclipse C1si; Nikon Corporation, Tokyo, Japan) or a fluorescence microscope (EVOSTM Auto 2; Invitrogen, WA, USA).

### 2.6 Wound healing assay

A549 cells were cultured in a six‐well plate and treated with different concentrations of Andro (2, 5 or 10 μM) in the presence and absence of TGF‐β1 as indicated. The wound healing assays were performed as described by Yang et al^19^ The wounds were observed using bright field microscopy (Leica, Weztlar, Germany), and multiple images were obtained at areas flanking the intersections of the wound and the marker lines after the scratch at 0, 6, 12 and 24 hours. Images were obtained for analysis using ImageJ software.

### 2.7 Measurement of intracellular ROS

Intracellular ROS generation was examined by dichlorodihydrofluorescein diacetate (DCFH‐DA; Beyotime Institute of Biotechnology, China). Briefly, A549 cells were seeded in six‐well black plates and treated as indicated. After the medium was removed, cells were incubated with 1 mL of DCFH‐DA solution (10 μM) at 37°C for 30 minutes. The dye solution was then removed, and the cells were trypsinized and centrifuged at 500 *g* for 5 minutes at 4°C. Finally, the cells were washed twice with PBS and kept in the dark on ice. The fluorescence intensity was measured with a microplate reader at 488 nm excitation and 525 nm emission wavelengths. Intracellular ROS production was also detected with a fluorescence microscope. After incubation for 20 minutes in the dark, the cells were washed three times with PBS, and the ROS level was examined with a fluorescence microscope.

### 2.8 Real‐time qPCR

Real‐time qPCR was performed as described previously.^17^ Briefly, total RNA was extracted from A549 cells using TRIzol reagent (Invitrogen Corporation, Carlsbad, CA, USA) and reverse‐transcribed into first‐strand cDNA using the PrimeScript RT reagent Kit with gDNA Eraser (Takara, Shiga, Japan). The mRNA levels were analysed with an iCyler iQ Real‐time PCR Detection System (Bio‐Rad Laboratories Inc.) using SsoFast EvaGreen Supermix (Bio‐Rad Laboratories Inc.) in a total volume of 15 μL. Relative levels of mRNA expression were normalized to 18s expression for each gene. The primers for real‐time qPCR assays are listed in Table 1.

**TABLE 1:** Primers used for real‐time qPCR

Gene	Sense primer(5′‐3′)	Antisense primer(5′‐3′)
Vimentin (h)	AGTCCACTGAGTACCGGAGAC	CATTTCACGCATCTGGCGTTC
Snail (h)	GCCTAGCGAGTGGTTCTTCT	TAGGGCTGCTGGAAGGTAAA
Slug (h)	GAGCATTTGCAGACAGGTCA	ACAGCAGCCAGATTCCTCAT
Collagen 1 (h)	GAGGGCCAAGACGAAGACATC	CAGATCACGTCATCGCACAAC
CCN‐2 (h)	GAGGAAAACATTAAGAAGGGCAA	CGGCACAGGTCTTGATGA
MMP‐2 (h)	AGTCTGAAGAGCGTGAAG	CCAGGTAGGAGTGAGAATG
MMP‐9 (h)	TGACAGCGACAAGAAGTG	CAGTGAAGCGGTACATAGG
18s	GCAATTATTCCCCATGAACG	GGCCTCACTAAACCATCCAA

### 2.9 Western blot

Western blot was performed as described previously.^17^ Briefly, the samples of A549 cells were homogenized in radioimmune precipitation assay lysis buffer with protease inhibitor and centrifuged to obtain the supernatants. The total protein concentration was determined by bicinchoninic acid (BCA) assay. Equal amounts (30 μg) of protein extracts from A549 cells were loaded into each lane onto 10% SDS‐polyacrylamide gels, transferred to polyvinylidene fluoride membranes (Millipore, Billerica, MA, USA) and incubated with appropriate primary antibodies overnight at 4°C. After reacting with HRP‐labelled secondary antibodies, the immunoreactive bands were visualized using an ECL chemiluminescent kit (Tiangen Biotech Co. Ltd, Beijing, China) and then scanned with Tanon‐5200 (Tanon Science & Technology Co., Ltd, Shanghai, China). The results were analysed by ImageJ.

### 2.10 Data and statistical analysis

Data were expressed as means ± SEM. Statistical analysis was performed with one‐way ANOVA. Differences between means were accept as significant at *P* < 0.05.

## 3 RESULTS

### 3.1 Effect of Andro on the viability of alveolar epithelial A549 cells

To investigate the cytotoxicity of Andro in AECs, human alveolar epithelial A549 cells were incubated with varying concentrations of Andro (0, 1, 2, 5, 10 and 20 μM) for 24 hours. CCK‐8 assay showed that Andro induced no significant cytotoxicity at the concentrations of 1, 2, 5 and 10 μM (Figure 1B), indicating that at the maximum concentration of 10 μM Andro did not affect A549 cell viability. Therefore, in the follow‐up experiments, the highest concentration of Andro we used was 10 μM.

### 3.2 Andro represses TGF‐β1‐induced EMT and EMT‐related transcription factors in A549 cells

**FIGURE 2:**
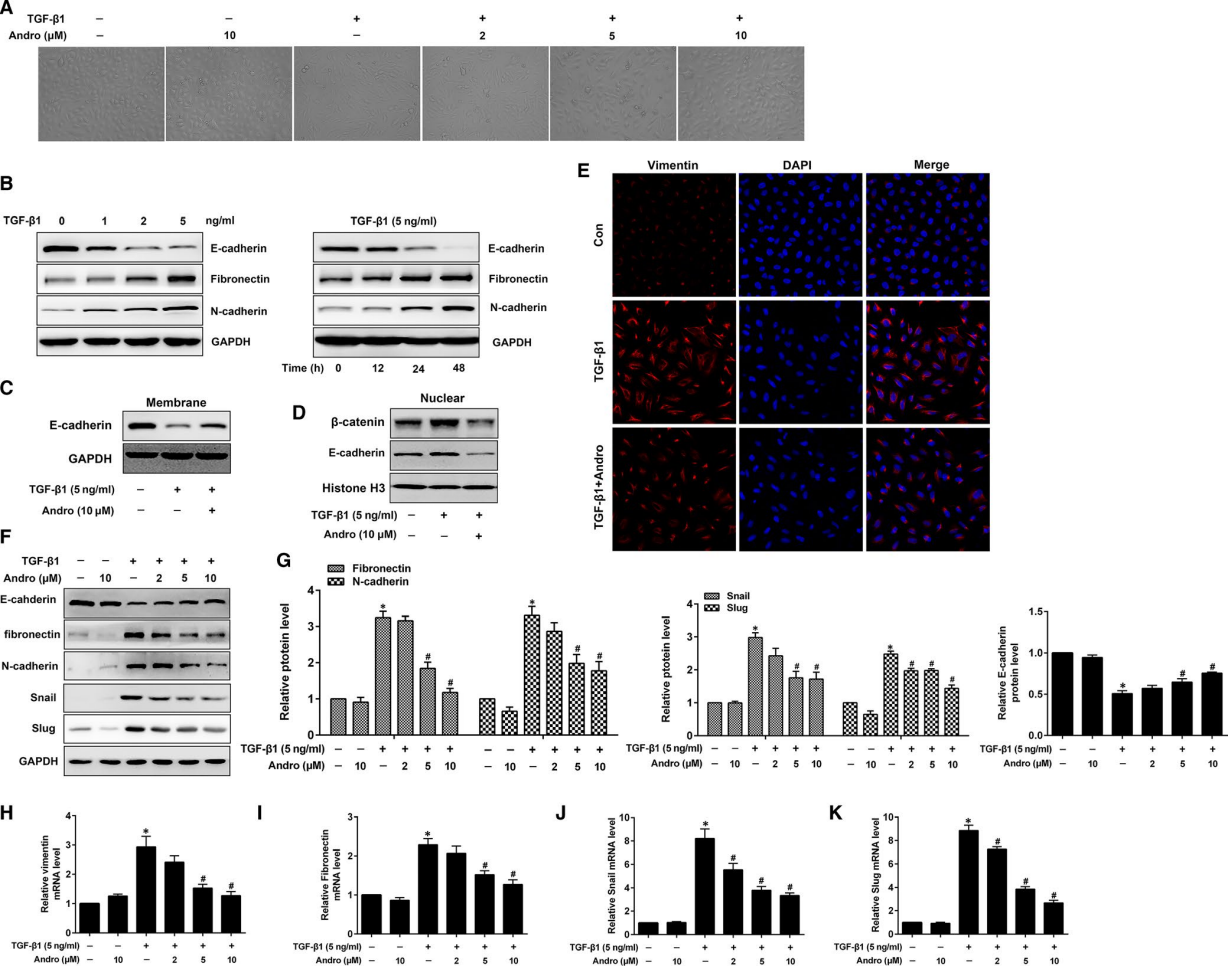
Andrographolide (Andro) suppressed TGF‐β1‐induced EMT and EMT‐related transcription factors in alveolar epithelial A549 cells. A549 cells were treated with TGF‐β1 (5 ng/mL) and different concentrations of Andro (2, 5, 10 μM) for 24 h. The cells stimulated with vehicle only served as controls. EMT and EMT‐related transcription factors were performed by examining (A) the morphological changes. Original magnification ×100. (B, F, G) the protein expression levels of E‐cadherin, fibronectin, N‐cadherin, Snail and Slug. (H‐K) the mRNA levels of vimentin, fibronectin, Snail and Slug. (C) A549 cells were treated with TGF‐β1 (5 ng/mL) and Andro (10 μM) for 24 h, and membrane was subjected to Western blot for E‐cadherin expression. (D) A549 cells were treated with TGF‐β1 (5 ng/mL) and Andro (10 μM) for 24 h, and nuclear lysates were subjected to Western blot for E‐cadherin or β‐catenin expression. (E) A549 cells were treated with TGF‐β1 (5 ng/mL) and Andro (10 μM) for 24 h, and vimentin was further examined by immunofluorescence. Original magnification ×200. The presented figures are representative data from at least three independent experiments. Data are presented as mean ± SEM, *P < 0.05 vs Con [TGF‐β1 (‐) and Andro (‐)]. ^#^ *P* < 0.05 vs TGF‐β1 only

We assessed whether Andro repressed TGF‐β1‐induced EMT in A549 cells. TGF‐β1‐induced EMT was confirmed by the surviving cell's phenotypic changes from an epithelial shape to a fibroblast‐like shape (Figure 2A). In accordance with these alterations, there were reduced E‐cadherin level and increased fibronectin, vimentin and N‐cadherin levels (Figure 2B,F‐I). Introduction of Andro inhibited TGF‐β1‐induced EMT, as measured by the alterations of morphology (Figure 2A) and EMT‐related biomarkers (Figure 2F‐I). This inhibiting effect of Andro on EMT is in a dose‐dependent manner. Likewise, our immunofluorescence studies also illustrated that Andro treatment down‐regulated vimentin expression as compared to TGF‐β1 alone treated cells (Figure 2E). Moreover, as Snail and Slug are considered to be the dominant transcriptional repressors of E‐cadherin, we then determined whether Andro mediated these EMT‐related transcription factors. The results demonstrated that the TGF‐β1‐induced up‐regulation of Snail and Slug was dose‐dependently reduced by Andro (Figure 2F,G,J,K). Additionally, we found that membrane‐bound E‐cadherin (Figure 2C) was significantly elevated, whereas nucleus E‐cadherin and β‐catenin were down‐regulated by Andro treatment (Figure 2D). Collectively, these data illustrated that Andro has a critical impact on inhibiting TGF‐β1‐induced EMT and on preserving the epithelial phenotype.

### 3.3 Andro regulates TGF‐β1‐induced EMT‐like cell behaviours in A549 cells

**FIGURE 3:**
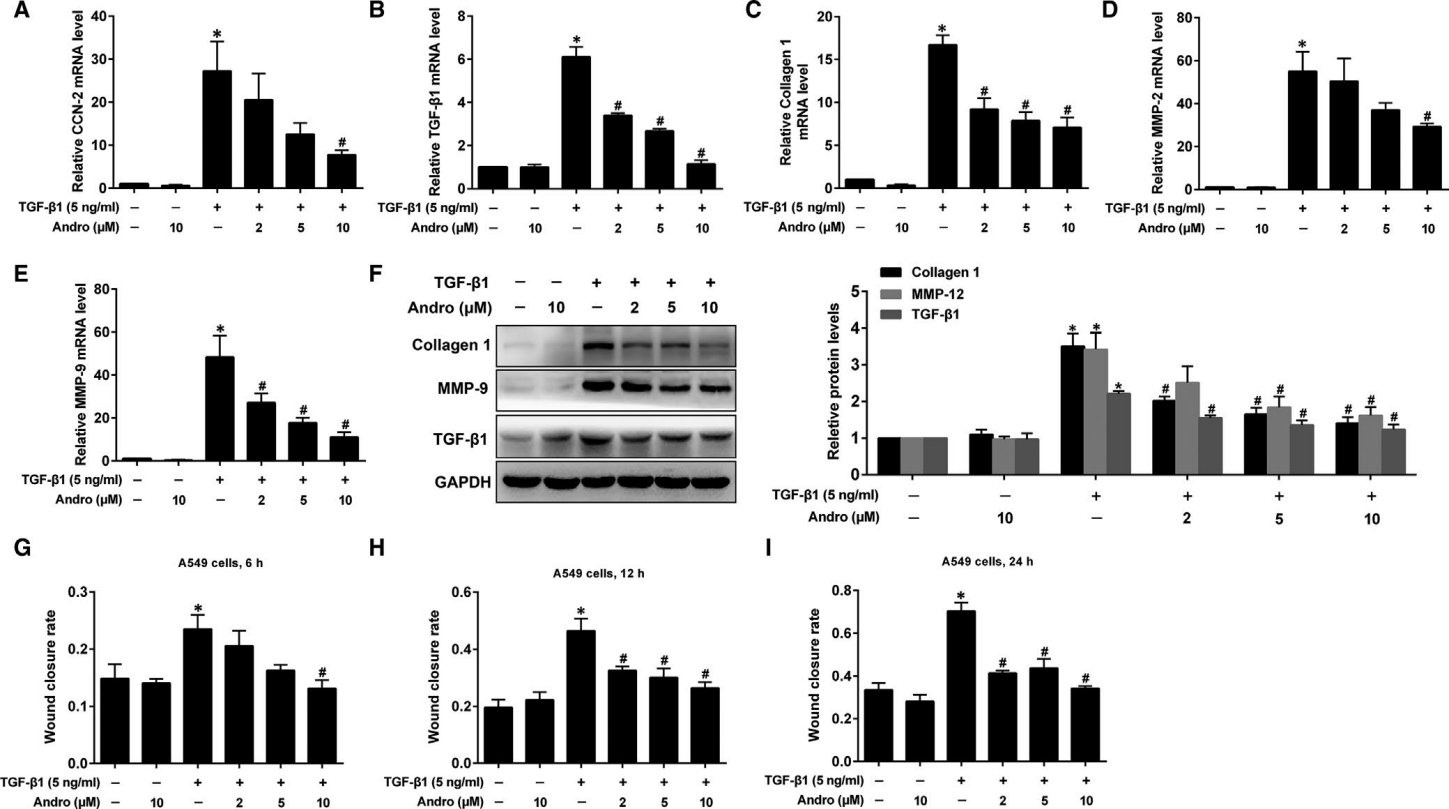
Andrographolide (Andro) regulated TGF‐β1‐induced EMT‐like cell behaviours in alveolar epithelial A549 cells. A549 cells were treated with TGF‐β1 (5 ng/mL) and different concentrations of Andro (2, 5, 10 μM) for 24 h. The mRNA levels of CCN‐2 (A), TGF‐β1 (B), collagen 1 (C), MMP‐2 (D) and MMP‐9 (E) were measured by real‐time qPCR. (F) The protein levels of collagen 1, MMP‐9 and TGF‐β1 were measured by Western blot. (G‐I) The wound closure was photographed at post‐scratching 0, 6, 12 and 24 h, and the wound closure rate at 6, 12 and 24 h, representing the migration rate, was detected by wound healing assay. The presented figures are representative data from at least three independent experiments. Data are expressed as mean ± SEM, *P < 0.05 vs control [TGF‐β1 (‐) and Andro (‐)]. ^#^ *P* < 0.05 vs TGF‐β1 only

Apart from phenotypic alterations, AECs also produce various pro‐fibrotic cytokines/growth factors such as TGF‐β1 and connective tissue growth factor (CTGF/CCN2), which further trigger EMT, and to aggravate lung fibrosis. Chen *et al* (2013) showed that TGF‐β1 also stimulated EMT‐like alteration in A549 cells.^5^ Furthermore, the injured AECs can also synthesize MMPs that promote the migration of fibroblasts and ECM remodelling in the pathogenesis of PF.^20^ Hence, we investigated whether Andro could repress TGF‐β1‐induced EMT‐like cell behaviours by repealing those pro‐fibrotic responses. TGF‐β1 prominently up‐regulated mRNA levels of CCN2, TGF‐β1, collagen 1, MMP‐2 and MMP‐9, and protein levels of collagen 1, MMP‐9 and TGF‐β1 in A549 cells, which were all significantly inhibited by Andro treatment (Figure 3A‐F). During EMT process, epithelial cells gain fibroblast‐like properties and display decreased intercellular adhesion and augmented motility. We demonstrated that Andro significantly repressed TGF‐β1‐induced migratory behaviour (Figure 3G‐I). These results indicate that Andro could suppress TGF‐β1‐induced EMT‐like cell behaviours.

### 3.4 Andro down‐regulates TGF‐β1‐activated Smad2/3 signalling pathways in alveolar epithelial A549 cells

**FIGURE 4:**
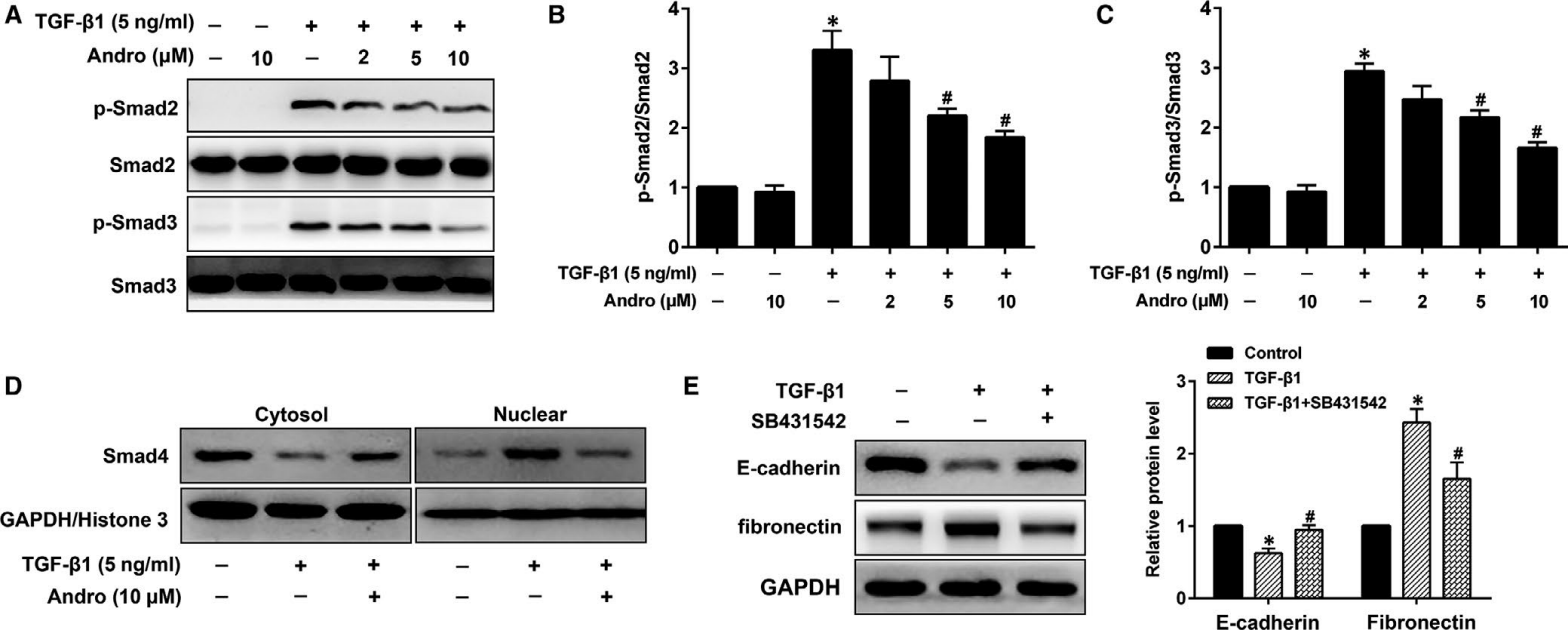
Andrographolide (Andro) down‐regulated TGF‐β1‐activated Smad2/3 signalling pathways in alveolar epithelial A549 cells. A549 cells were treated with TGF‐β1 (5 ng/mL) and different concentrations of Andro (2, 5, 10 μM) for 24 h. (A) The protein levels of p‐Smad2 and p‐Smad3 were measured by Western blot. (B, C) Expression of p‐Smad2 and p‐Smad3 was normalized to Smad2 and Smad3 levels, respectively. The presented figures are representative data from at least three independent experiments. Data are expressed as mean ± SEM, *P < 0.05 vs control [TGF‐β1 (‐) and Andro (‐)]. ^#^ *P* < 0.05 vs TGF‐β1 only. (D) A549 cells were treated with TGF‐β1 (5 ng/mL) and/or10 μM Andro for 24 h, and cytosolic and nuclear lysates were subjected to Western blot for Smad4. (E) A549 cells were cultured with SB431542 in the presence of TGF‐β1 for 24 h. The protein levels of E‐cadherin and fibronectin were detected using Western blot. The presented figures are representative data from at least three independent experiments. Data are presented as mean ± SEM, *P < 0.05 vs Con [TGF‐β1 (‐) and SB431542 (‐)]. ^#^ *P* < 0.05 vs TGF‐β1 only

The ability of TGF‐β1/Smad signalling stimulate EMT in AECs is well documented.^21^ We therefore investigated whether Andro attenuated TGF‐β1‐induced EMT through inactivation of TGF‐β1‐Smad2/3 signalling pathway. Western blot assay was used to further investigate the intracellular signal transduction mechanism, and found that Andro notably reduced TGF‐β1‐induced phosphorylation of Smad2 (Ser465/Ser467) and Smad3 (Ser423/Ser425) in A549 cells (Figure 4A‐C). And Andro treatment obviously blocked TGF‐β1‐induced shuttling of Smad4 from cytosol to nucleus (Figure 4D). To further investigate the decisive role of Smad2/3 in TGF‐β1‐induced EMT, an ALK5 inhibitor (TGF‐β type Ⅰ inhibitor) SB431542 was used in A549 cells. As expected, the induction of EMT caused by TGF‐β1 was significantly ameliorated by SB431542 treatment (Figure 4E). Taken together, these data suggest that the inhibition of Smad2/3 by Andro is involved in the attenuation of EMT in epithelial A549 cells.

### 3.5 Andro down‐regulates TGF‐β1‐activated Erk1/2 signalling in A549 cells

**FIGURE 5:**
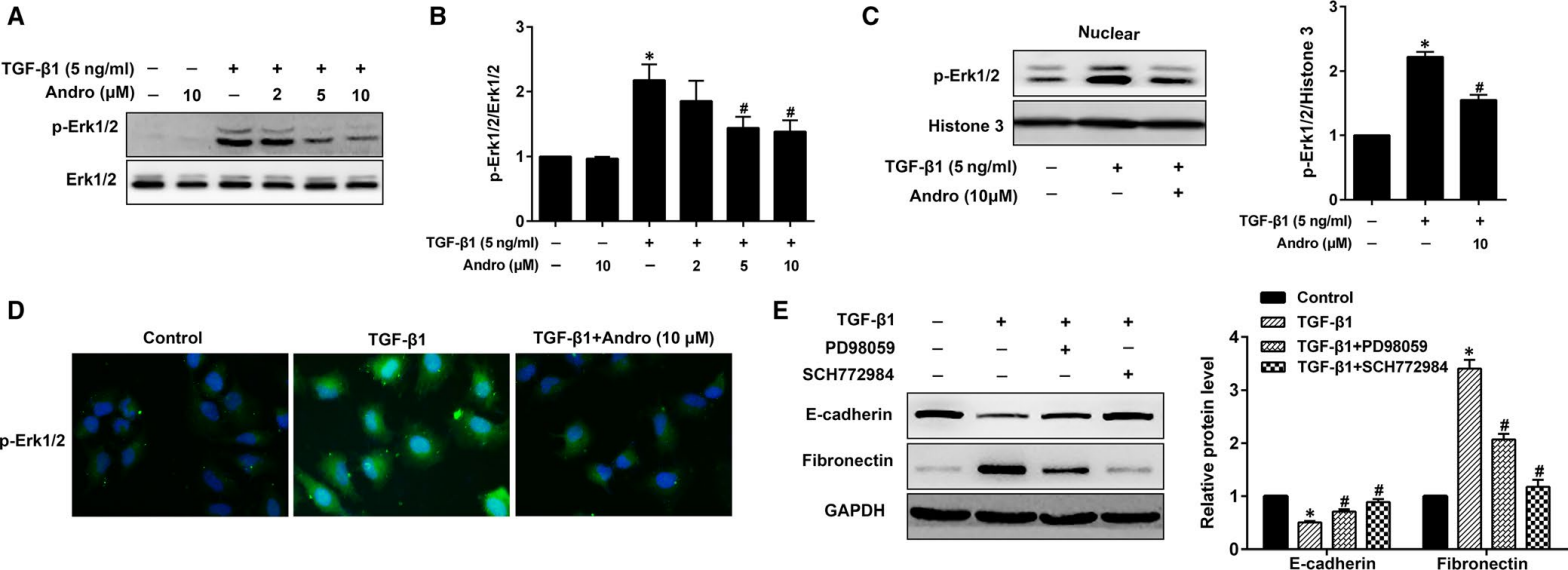
Andrographolide (Andro) down‐regulated TGF‐β1‐activated Erk1/2 signalling in alveolar epithelial A549 cells. (A) A549 cells were treated with TGF‐β1 (5 ng/mL) and different concentrations of Andro (2, 5, 10 μM) for 24 h. The protein level of p‐Erk1/2 was measured by Western blot. (B) Expression of p‐Erk1/2 was normalized to Erk1/2 level. The presented figures are representative data from at least three independent experiments. (C) A549 cells were treated with TGF‐β1 (5 ng/mL) and Andro (10 μM) for 24 h, and nuclear lysates were subjected to Western blot for p‐Erk1/2. The presented figures are representative data from at least three independent experiments. Data are presented as mean ± SEM, *P < 0.05 vs control [TGF‐β1 (‐) and Andro (‐)]. ^#^ *P* < 0.05 vs TGF‐β1 only. (D) A549 cells were treated with TGF‐β1 (5 ng/mL) and Andro (10 μM) for 24 h, and p‐Erk1/2 was further examined by immunofluorescence. Original magnification ×200. (E) A549 cells were cultured with PD98059 or SCH772984 in the presence of TGF‐β1 for 24 h. The protein levels of E‐cadherin and fibronectin were detected using Western blot. The presented figures are representative data from at least three independent experiments. Data are presented as mean ± SEM, *P < 0.05 vs control [TGF‐β1 (‐), PD98059 (‐) and SCH772954 (‐)]. ^#^ *P* < 0.05 vs TGF‐β1 only

TGF‐β1 is also known to induce EMT by Smad‐independent pathways including Erk1/2.^11^ We next investigated whether Andro mediated TGF‐β1‐induced EMT via the regulation of Erk1/2 activation. As shown in Figure 5A,B, TGF‐β1‐treated cells showed increased phosphorylated Erk1/2 in comparison with control cells. However, Andro treatment markedly reduced the TGF‐β1‐induced Erk1/2 phosphorylation, indicating that Andro antagonized TGF‐β1‐mediated Erk1/2 signalling to repress EMT. We also determined whether Andro affected TGF‐β1‐induced Erk1/2 nuclear translocation. Western bolt analysis demonstrated that TGF‐β1‐induced increase of Erk1/2 nuclear translocation was inhibited by Andro treatment (Figure 5C). Likewise, the immunofluorescence study also showed that TGF‐β1‐induced Erk1/2 phosphorylation and nuclear translocation were down‐regulated by Andro (Figure 5D). To further confirm the important role of Erk1/2 in TGF‐β1‐induced EMT, both SCH 772984 (a specific Erk1/2 inhibitor) and PD98059 (a specific MAPK inhibitor) were used in A549 cells. As expected, the Erk1/2‐inhibited A549 cells were dramatically insensitive to TGF‐β1‐induced EMT (Figure 5E). These data indicate that Andro repressed TGF‐β1‐induced activation of Erk1/2 signalling to inhibit EMT in AECs.

### 3.6 Andro reduces TGF‐β1‐induced ROS generation and modulates the oxidant/antioxidant imbalance in A549 cells

**FIGURE 6:**
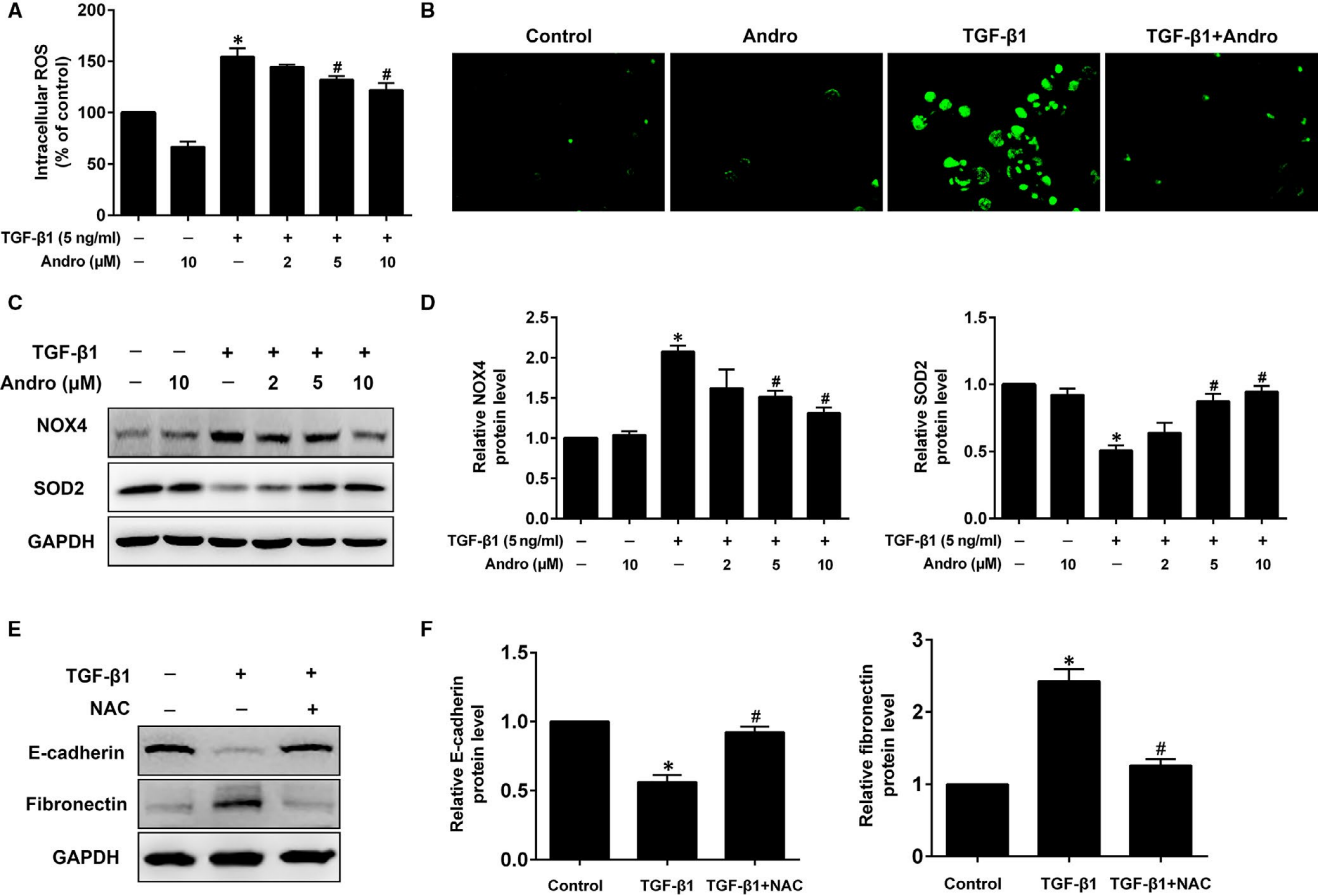
Andrographolide (Andro) reduced TGF‐β1‐induced intracellular ROS production and modulated the oxidant/antioxidant imbalance in alveolar epithelial A549 cells. A549 cells were treated with TGF‐β1 (5 ng/mL) and different concentrations of Andro (2, 5, 10 μM) for 24 h. (A) The intracellular ROS was determined using a fluorescence microplate according to the ROS assay kit. Original magnification ×200. (B) Fluorescence intensity was measured using fluorescence microscope. (C, D) Western blot was used to detect NOX4 and SOD2 expressions. The presented figures are representative data from at least three independent experiments. Data are presented as mean ± SEM, *P < 0.05 vs Con (TGF‐β1 [‐] and Andro [‐]). ^#^ *P* < 0.05 vs TGF‐β1 only. (E, F) A549 cells were treated with TGF‐β1 and the ROS scavenger N‐Acetyl‐L‐cysteine (NAC) for 24 h. Western blot was used to detect E‐cadherin and fibronectin expressions. The presented figures are representative data from at least three independent experiments. Data are expressed as mean ± SEM, *P < 0.05 vs control [TGF‐β1 (‐) and NAC (‐)]. ^#^ *P* < 0.05 vs TGF‐β1 only

Since ROS can mediate many cellular functions including EMT, we investigated whether Andro affects ROS synthesis during TGF‐β1‐induced EMT. TGF‐β1 dramatically increased ROS levels in A549 cells, which was reduced by Andro intervention (Figure 6A). Similarly, fluorescence microscopy revealed that TGF‐β1 significantly enhanced DCF intensity, and Andro repressed this effect to near basal levels (Figure 6B). ROS can be induced by the imbalanced oxidant/antioxidant enzymes, such as enhanced NADPH oxidase 4 (NOX4) activity and reduced SOD2 levels. We, therefore, investigated whether the inhibition of ROS production by Andro is due to the regulation of the oxidant/antioxidant imbalance. Western blot analysis showed that TGF‐β1 significantly enhanced NOX4 protein expression and decreased SOD2 protein levels; however, these changes were ameliorated by Andro treatment (Figure 6C,D), further indicating the antioxidant role of Andro. Further, we demonstrated that treatment of A549 cells with the ROS scavenger N‐Acetyl‐L‐cysteine (NAC) significantly suppressed TGF‐β1‐induced up‐regulation of fibronectin and down‐regulation of E‐cadherin (Figure 6E,F), suggesting that ROS is involved in TGF‐β1‐induced EMT in A549 cells. These findings indicate that Andro prevents TGF‐β1‐induced oxidative stress, which is closely linked to EMT.

### 3.7 Andro activates Sirt1/FOXO3 pathway in TGF‐β1‐induced EMT in A549 cells

**FIGURE 7:**
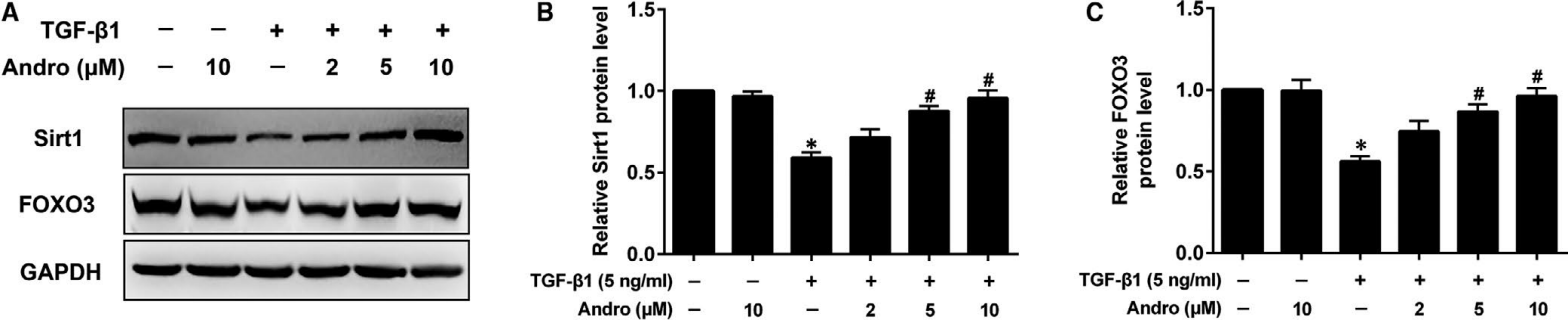
Andrographolide (Andro) activated Sirt1/FOXO3 signalling in TGF‐β1‐induced EMT in alveolar epithelial A549 cells. A549 cells were treated with TGF‐β1 (5 ng/mL) and different concentrations of Andro (2, 5, 10 μM) for 24 h. (A) The protein levels of Sirt1 and FOXO3 were measured by Western blot. (B, C) Densitometric analysis of Sirt1 and FOXO3 in the immunoblots using GAPDH as the internal reference. The presented figures are representative data from at least three independent experiments. Data are expressed as mean ± SEM, *P < 0.05 vs control [TGF‐β1 (‐) and Andro (‐)]. ^#^ *P* < 0.05 vs TGF‐β1 only

Sirt1/FOXO3 signalling can regulate oxidative stress and EMT.^10^, ^22^ We, therefore, determined Sirt1 and FOXO3 expressions in the absence or presence of Andro in response to TGF‐β1 stimulation. The protein expressions of Sirt1 and FOXO3 were significantly decreased in TGF‐β1‐treated A549 cells. Expression levels of Sirt1 and FOXO3 were elevated to near baseline by Andro treatment (Figure 7). These data suggest that Andro activated Sirt1/FOXO3‐mediated anti‐oxidative stress pathway to suppress TGF‐β1‐induced EMT in A549 cells.

## 4 DISCUSSION

Epithelial‐mesenchymal transition has been cumulatively considered as a mechanism for fibrotic diseases, and it plays a critical role in the development of PF.^8^ Because TGF‐β1 is a primary inducer of PF, studies on TGF‐β1‐induced EMT in human AECs have a great significance for understanding the mechanism of PF. In the present study, we demonstrated that Andro attenuated TGF‐β1‐induced EMT and EMT‐like cell behaviours in alveolar epithelial A549 cells by inhibiting both Smad2/3 signalling pathways and Erk1/2 signalling pathway. Furthermore, Andro also activated Sirt1/FOXO3‐mediated anti‐oxidative stress pathway to suppress TGF‐β1‐induced EMT in A549 cells. This is the first study to demonstrate the direct effects of Andro on TGF‐β1‐induced EMT within AECs, which may provide a novel therapy for EMT‐related diseases.

Pulmonary fibrosis is characterized by extravagant accumulation of fibroblasts/myofibroblasts and ECM in the lung that destroys its architecture and function.^23^ Although the pathogenesis of PF is largely unknown, epithelial cells, in particular type II AECs, have been identified as one of the critical players in this disease.^23^ Primary human type II AECs undergo a TGF‐β1‐dependent EMT and thus acquire the ability to produce collagens, promoting PF.^8^, ^24^ Hence, TGF‐β1 was widely used as a stimulator to trigger EMT in human type II AECs.^25^

Alveolar epithelial cells, for example A549 cells, in response to TGF‐β1, can also undergo that EMT and differentiate into fibroblasts and myofibroblasts.^11^ Recent studies have shown that Andro attenuated silica‐induced EMT in mice,^18^ and antagonized cigarette smoke‐induced EMT and pulmonary dysfunction through inhibiting HOTAIR.^26^ Nevertheless, the direct effects of Andro on TGF‐β1‐induced EMT in AECs remain uncertain. Here, we found that Andro treatment significantly suppressed TGF‐β1‐induced EMT process in A549 cells, as demonstrated by the alterations of morphology and differentiation markers. During EMT process, epithelial cells gain fibroblast‐like properties and thus exhibit decreased intercellular adhesion and augmented motility.^27^ Likewise, TGF‐β1 can also accelerate EMT and subsequently augment the migratory capacity of epithelial cells in a frustrated effort of lung repair. The suppression of Andro on the migration of TGF‐β1‐stimulated A549 cells further suggests the inhibitory effect of Andro on EMT, and also provides a sound explanation for its role in enhancing cisplatin‐mediated anticancer effects on lung cancer cells^28^ and reducing breast cancer growth and metastasis.^29^ Apart from phenotypic alterations, epithelial cells exposed to TGF‐β1 also experience functional transition that is characterized by production of a variety of pro‐fibrotic mediators and ECM molecules, which in turn further induces EMT and to deteriorate PF.^30^, ^31^ They also synthesize a large of MMPs that promote the migration of fibroblasts and ECM remodelling during the development of PF. Accordingly, the expressions of CCN2, TGF‐β1, collagen 1, MMP‐2 and MMP‐9 were all increased after TGF‐β1 stimulation, which were suppressed by Andro treatment, suggesting the suppressive role of Andro in EMT‐like cell behaviours. The above findings indicate that the anti‐fibrotic effects of Andro are, at least in part, due to its disturbance with the TGF‐β1‐mediated EMT. Our results are partially consistent with a previous study by Kayastha *et al* who reported that Andro suppressed TGF‐β2‐induced EMT in lens epithelial FHL 124 cells.^14^ To our knowledge, this is the first time to demonstrate that Andro could attenuate the EMT induced by TGF‐β1, the most critical inductor of EMT in PF, in AECs. Since EMT of AECs is an important source of ECM during PF, the inhibition of TGF‐β1‐induced EMT participates in the protective effects of Andro against PF.

The loss of E‐cadherin is identified as a critical event during the pathogenesis of EMT.^32^ It appears that the up‐regulation of E‐cadherin by Andro was due to the inhibition of Snail family members (Snail and Slug), two important endogenous repressors of E‐cadherin. Moreover, E‐cadherin is a key transmembrane protein, which can interact with β‐catenin to mediate cell adhesion.^33^ Hence, we speculated that the overall enhancement in E‐cadherin level by Andro attributes to the up‐regulation of membrane‐bound E‐cadherin. In accordance with our hypothesis, Andro remarkably decreased E‐cadherin expression in cytosolic and nuclear fractions whereas elevated its expression in the cell membrane fraction. Our speculation was also confirmed by the results that Andro‐treated A549 cells also had an increase in the E‐cadherin‐binding protein, β‐catenin in the membrane, and decreased β‐catenin level in the nuclear.

The mechanism by which Andro inhibits TGF‐β1‐induced EMT in AECs remains obscure. The inhibitory effect of Andro on TGF‐β1‐induced EMT indicates the probability that Andro may modulate the key molecules in the TGF‐β1 signalling pathway. Smads are crucial intracellular mediators of TGF‐β1‐induced EMT.^11^ Moreover, activation of Smad2 and/or Smad3 caused increased PF in rats or C57BL/6J mice.^27^ Apart from this canonical signalling, activation of some non‐Smad signalling by TGF‐β1, including Erk1/2, also promotes EMT process. Studies have shown that Erk1/2 signalling is essential for EMT induced by TGF‐β1, which eventually contributes to the pathobiology of fibrosis.^11^, ^34^ In this study, we found that treatment with A549 cells with Andro significantly inhibited TGF‐β1‐induced Smad2/3 and Erk1/2 phosphorylation and nuclear translocation, implying the Andro's suppressive effect on EMT is linked to the suppression of TGF‐β1‐activated Smad2/3 and Erk1/2 signalling pathways. Moreover, the suppression of Smad2/3 and Erk1/2 using their pharmacological inhibitors memorably ameliorated TGF‐β1‐induced EMT in A549 cells, further illustrating the significance of Andro in antagonizing TGF‐β1 signalling. Collectively, these results suggest that Andro ameliorates TGF‐β1‐induced EMT in AECs through inhibiting Smad2/3 and Erk1/2 signalling pathways.

Additionally, ROS are the primary contributors to oxidative stress and are essential for TGF‐β1‐induced EMT by altering gene expression, cytoskeletal rearrangement and cell migration in epithelial cells including AECs.^35^ It has been reported that Andro could significantly inhibit the oxidized low‐density lipoprotein (oxLDL)‐induced ROS production.^36^ However, whether Andro mediated ROS generation in TGF‐β1‐induced EMT of A549 cells is unknown. Our study showed that Andro significantly reduced TGF‐β1‐induced ROS production, which has been clarified in this study to be closely associated with EMT in A549 cells. NOX family is a critical source of ROS influencing cell migration and fibronectin expression in EMT.^37^ NOX4 facilities the diminution of molecular oxygen to superoxide to induce ROS production, while SOD2 negatively regulates NOX4 and acts as a critical antioxidant enzyme.^35^ When oxidant/antioxidant imbalance occurs, excess ROS are synthesized resulting in a series of pathological events, including EMT. The present study demonstrated that Andro also attenuates TGF‐β1‐induced up‐regulation of NOX4 and down‐regulation of SOD2. This suggests that Andro may inhibit TGF‐β1‐induced EMT through inhibiting ROS pathway.

FOXO3 is a transcription factor that is involved in diverse physiological and pathological processes, including oxidative stress, proliferation, apoptosis, immunity and differentiation.^38^ FOXO3 inactivation positively correlates with cumulating of collagens and up‐regulation of α‐SMA in the renal fibrosis.^39^ Mice with FOXO3 gene knockout showed enhanced susceptibility to BLM challenge, as well as increased fibrosis, deterioration of lung function, and enhanced mortality.^40^ Moreover, overexpression of FOXO3 inhibited TGF‐β1‐induced EMT in A549 cells.^10^ Sirt1, a NAD‐dependent deacetylation enzyme, plays an important role in many pathophysiological processes, including inflammation, oxidative stress and EMT.^41^ Sirt1 can stimulate the expression and transcriptional activity of FOXO3, which then promotes the expressions of its target genes including SOD in various cells.^22^ Sirt1 also increases the ability of FOXO3 to trigger cell cycle arrest and counteract with the ROS induced oxidative stress. Previous study has shown that Sirt1 was markedly reduced in BLM‐induced PF, and treatment with Sirt1 activator attenuated the BLM‐induced EMT in mice.^42^, ^43^ Sirt1 expression was also decreased in A549 cells after TGF‐β1 treatment, and activated Sirt1 suppressed TGF‐β1‐induced decrease of ACE2 in A549 cells, which has significant protective effects against PF.^8^ Moreover, sirt1 up‐regulation not only attenuates Smad2/3 transactivation,^44^ but also inhibited TGF‐β1‐induced EMT in alveolar epithelial cells.^45^ Hence, activating Sirt1/FOXO3 signalling is an important therapeutic target for PF. Interestingly, Sirt1 and FOXO3 expressions were remarkably elevated in Andro‐treated cells in comparison with the TGF‐β1 alone treated cells. Taken together, Andro also protects AECs from EMT through activating Sirt1/FOXO3‐mediated anti‐oxidative stress signalling pathway.

In addition to mediating EMT, Smad2/3, specific Sirt1 targets, also regulate oxidative stress in cells. For instance, Smad3 overexpression can initiate oxidative stress and protein degradation,^46^ while Smad3 silencing decreased Nox4 expression and prevented oxidative stress‐induced damage.^47^ Additionally, inhibition of Smad3 expression using SB431542 reversed TGF‐β1‐induced decrease in FOXO3 in A549 cells.^10^ Similarly, Erk1/2 pathway, another specific SIRT1 target, is also involved in the regulation of FOXO3, and thus reduced oxidative stress and EMT.^48^ These findings indicate that the down‐regulation of Smad2/3 and Erk1/2 by Andro might be also involved in the regulation of FOXO3 and the alleviation of oxidative stress in A549 cells. In return, the reduction of oxidative stress by Andro may contribute to the inactivation of Smad2/3 and Erk1/2 signalling pathways as well, since inhibition of ROS production by NAC could inhibit the phosphorylation of Smad2/3 and Erk1/2.

In summary, we demonstrate firstly that Andro attenuates TGF‐β1‐induced EMT in alveolar epithelial A549 cells by suppressing TGF‐β1‐activated Smad2/3 and Erk1/2 signalling pathways. Andro also decreases ROS generation in TGF‐β1‐induced EMT process. We propose that Andro is a promising pharmacological tool for the treatment of PF.

## CONFLICTS OF INTEREST

There are no conflicts of interest to declare.

## AUTHOR CONTRIBUTION

**Jingpei Li:** Conceptualization (lead); Funding acquisition (lead); Investigation (equal); Methodology (equal); Project administration (equal); Writing‐original draft (equal); Writing‐review & editing (equal). **Jun Liu:** Data curation (lead); Formal analysis (lead); Writing‐original draft (equal); Writing‐review & editing (equal). **Weifeng Yue:** Investigation (equal); Methodology (equal); Writing‐original draft (equal); Writing‐review & editing (equal). **Ke Xu:** Investigation (equal); Methodology (equal). **Weipeng Cai:** Data curation (equal); Formal analysis (equal). **Fei Cui:** Investigation (equal); Methodology (equal). **Zhuoyi Li:** Investigation (equal); Methodology (equal). **Wei Wang:** Investigation (equal); Methodology (equal). **Jianxing He:** Conceptualization (equal); Project administration (equal); Supervision (lead); Writing‐original draft (equal); Writing‐review & editing (equal).

## Data Availability

Data of this manuscript are available from the corresponding author upon reasonable request.
